# The use of temozolomide in paediatric metastatic phaeochromocytoma/paraganglioma: A case report and literature review

**DOI:** 10.3389/fendo.2022.1066208

**Published:** 2022-11-09

**Authors:** Calum Urquhart, Ben Fleming, Ines Harper, Luigi Aloj, Ruth Armstrong, Liz Hook, Anna-May Long, Claire Jackson, Ferdia A. Gallagher, Mary A. McLean, Patrick Tarpey, Vasilis Kosmoliaptsis, James Nicholson, A. Emile J. Hendriks, Ruth T. Casey

**Affiliations:** ^1^ Department of Diabetes and Endocrinology, Cambridge University Hospitals NHS Foundation Trust, Cambridge, United Kingdom; ^2^ Department of Radiology, Cambridge University Hospitals NHS Foundation Trust, Cambridge, United Kingdom; ^3^ Department of Nuclear Medicine, Cambridge University Hospitals NHS Foundation Trust, Cambridge, United Kingdom; ^4^ Department of Radiology, University of Cambridge, Cambridge, United Kingdom; ^5^ Department of Clinical Genetics, Cambridge University Hospitals NHS Foundation Trust, Cambridge, United Kingdom; ^6^ Department of Pathology, Cambridge University Hospitals NHS Foundation Trust, Cambridge, United Kingdom; ^7^ Department of Paediatric Surgery, Cambridge University Hospitals NHS Foundation Trust, Cambridge, United Kingdom; ^8^ Department of Surgery and NIHR Biomedical Research Centre, University of Cambridge and Cambridge University Hospitals NHS Foundation Trust, Cambridge, United Kingdom; ^9^ Department of Paediatric Oncology, Cambridge University Hospitals NHS Foundation Trust, Cambridge, United Kingdom; ^10^ Department of Paediatrics, University of Cambridge, Cambridge, United Kingdom; ^11^ Department of Paediatric Diabetes and Endocrinology, Cambridge University Hospitals NHS Foundation Trust, Cambridge, United Kingdom; ^12^ Department of Medical Genetics, University of Cambridge, Cambridge, United Kingdom

**Keywords:** paraganglioma, pheochromocytoma, temozolomide, succinate dehydrogenase (SDH), SDHB = SDH enzyme complex subunit B

## Abstract

There is increasing evidence to support the use of temozolomide therapy for the treatment of metastatic phaeochromocytoma/paraganglioma (PPGL) in adults, particularly in patients with *SDHx* mutations. In children however, very little data is available. In this report, we present the case of a 12-year-old female with a SDHB-related metastatic paraganglioma treated with surgery followed by temozolomide therapy. The patient presented with symptoms of palpitations, sweating, flushing and hypertension and was diagnosed with a paraganglioma. The primary mass was surgically resected six weeks later after appropriate alpha- and beta-blockade. During the surgery extensive nodal disease was identified that had been masked by the larger paraganglioma. Histological review confirmed a diagnosis of a metastatic SDHB-deficient paraganglioma with nodal involvement. Post-operatively, these nodal lesions demonstrated tracer uptake on ^18^F-FDG PET-CT. Due to poor tumour tracer uptake on ^68^Ga-DOTATATE and ^123^I-MIBG functional imaging studies radionuclide therapy was not undertaken as a potential therapeutic option for this patient. Due to the low tumour burden and lack of clinical symptoms, the multi-disciplinary team opted for close surveillance for the first year, during which time the patient continued to thrive and progress through puberty. 13 months after surgery, evidence of radiological and biochemical progression prompted the decision to start systemic monotherapy using temozolomide. The patient has now completed ten cycles of therapy with limited adverse effects and has benefited from a partial radiological and biochemical response.

## Case description

A 12-year-old girl presented to a clinic appointment with a two-year history of sweating, intermittent flushing and occasional headaches. After monitoring her heart rate using a wearable device, she and her parents noted tachycardia even at rest, with a heart rate ranging from 120 to 145 beats per minute.

Upon presentation, she was tachycardic, hypertensive (207/184 mmHg) and a left parasternal heave was palpable. She was noted by the assessing clinician to be of short stature, with a current height of 136.4 cm (0.4^th^ centile) while her target height was near the 70^th^ centile. In the absence of any pubertal development, she was also diagnosed with delayed puberty.

## Diagnosis, treatment, and outcomes

**Figure 1:**
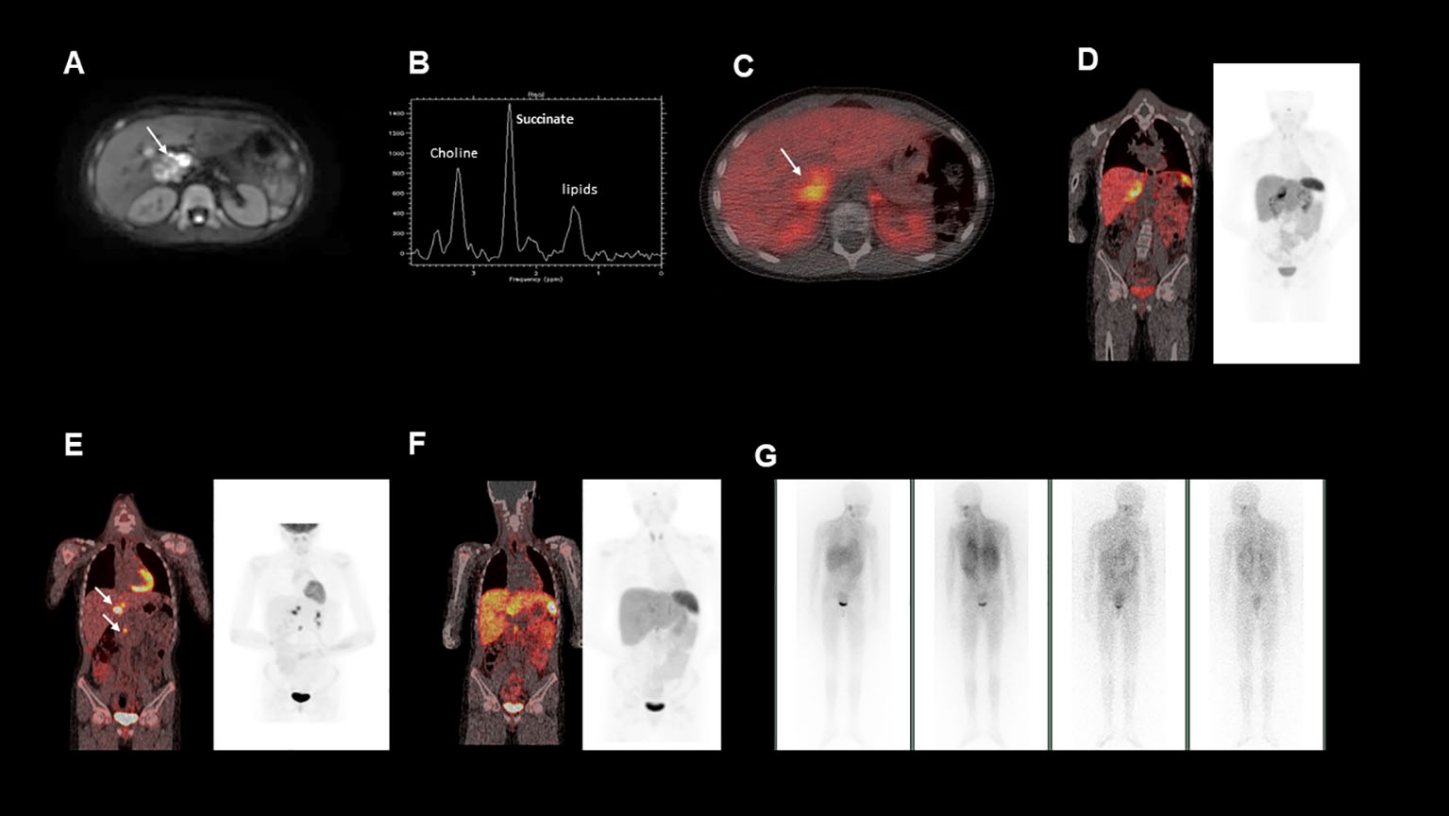
**(A)**, This is an axial diffusion-weighted MRI image showing a right sided heterogeneous suprarenal mass. **(B)**, MRI spectroscopy acquired from a voxel placed within the right suprarenal mass demonstrated a high succinate concentration. **(C**, **D)**, show fused axial and coronal images from a ^68^Ga-DOTATATE PET/CT scan showing heterogeneous uptake in the right suprarenal mass. **(E)**, Fused coronal image from a post operative ^18^FDG-PET/CT, demonstrating FDG avid nodal metastases near the liver hilum and aortocaval region **(F)**, Fused coronal image from a post operative ^68^Ga-DOTATATE PET/CT, demonstrating minimal uptake in the identified nodal metastases **(G)**, Post-operative ^123^I-MIBG whole body scan, which showed no significant MIBG uptake in the known metastatic deposits.

A magnetic resonance imaging (MRI) scan of the abdomen was performed, which showed a 6 cm x 3 cm right-sided suprarenal mass abutting the liver, inferior vena cava and hilum, but without features of direct invasion on imaging (**Figure 1A**). An echocardiogram showed left ventricular hypertrophy, indicating that the patient’s hypertension was likely longstanding. The patient was started on an alpha- and beta-blockade with phenoxybenzamine and atenolol respectively and admitted to manage her hypertension. Her ongoing care was subsequently transferred to our tertiary centre.

To further characterise the suprarenal mass, a ^68^Ga-DOTATATE positron emission tomography (PET-CT) was performed. The mass demonstrated heterogenous ^68^Ga-DOTATATE uptake (**Figures 1C**, **D**), in keeping with a somatostatin receptor positive tumour such as a paraganglioma or phaeochromocytoma, with the former diagnosis suspected in this case. In addition, the patient’s urinary metanephrine concentration was 64591 nmol/l, significantly elevated above the reference range.

Surgical removal of the mass was planned. Magnetic resonance (MR) spectroscopy was performed (**Figure 1B**), demonstrating that the tumour had a high succinate concentration in keeping with a succinate dehydrogenase (SDH)-deficient paraganglioma (1). Due to the increased risk of malignant or aggressive behaviour from an SDH-deficient paraganglioma, the agreed pre-operative plan was to aim for complete surgical resection.

Six weeks after the patient’s presentation, an open resection of the mass was performed. As the mass was adherent to the gallbladder and liver, a cholecystectomy and partial caudate lobe resection were necessary. Additionally, the mass was found to be invading the inferior vena cava, requiring resection and reconstruction of the retro-hepatic vena cava with a deceased donor aortic graft. Intra-operatively, extensive nodal disease (not identified on pre-operative imaging) was noted including para-aortic and hilar nodes; some of the latter involved the first order bile ducts at the hilar plate which were not amenable to resection.

Histological analysis of the tumour confirmed an *SDHB*-deficient paraganglioma. Extensive lymph node involvement was also shown, confirming the diagnosis of a malignant SDH-deficient paraganglioma. Post-operatively a restaging ^18^F-fluorodeoxyglucose (FDG) PET-CT was performed (**Figure 1E**). This demonstrated FDG-avid deposits in the porta hepatis and the aortocaval region suspicious for metastatic nodal disease. Debulking surgery was discussed but was not believed to be feasible without major liver resection and attendant significant morbidity. At that time, the patient was asymptomatic, the tumour volume was low, and plasma metanephrines had almost normalised (**Table 1**). After discussion at a multi-disciplinary team meeting (MDT), close surveillance was recommended over systemic treatment to allow the patient to advance through puberty and adolescence, a critical period of growth and physical, emotional and social development.

**Table 1:** Table showing concentration of plasma metanephrine and plasma normetanephrine at various timepoints.

Date	Plasma metanephrine (pmol/l)	Plasma normetanephrine (pmol/l)	Notes
04/09/2020			Operation to remove tumour
**23/09/2020**	<180	1346	3 weeks post operation
**04/01/2021**	<180	2888	4 months post operation
17/02/2021			Doxazosin 1mg od restarted
**25/03/2021**	<180	3128	Doxazosin increased to 4mg od
**25/06/2021**	<180	3641	9 months post operation
**04/10/2021**	301	5268	Prior to 1^st^ cycle of temozolomide
**04/02/2022**	<180	3485	Prior to 5^th^ cycle of temozolomide
**14/04/2022**	<180	3787	Prior to 7^th^ cycle of temozolomide
**28/07/2022**	<180	2474	After 10^th^ cycle of temozolomide

**Reference range for plasma metanephrine:** <600 pmol/l - catecholamine secreting tumour unlikely; between 600 and 900 pmol/l – equivocal; >900 pmol/l - high likelihood of catecholamine secreting tumour.

**Reference range for plasma normetanephrine:** <1000 pmol/l - catecholamine secreting tumour unlikely; between 1000 and 2500 pmol/l – equivocal; >2500 pmol/l - high likelihood of catecholamine secreting tumour.

Bold was used to show dates for which biochemical values were available; non-bold text was used for important dates without biochemical values.

Also included are the dates of the patient’s operation, and the restarting of alpha-blockade.

**Figure 2:**
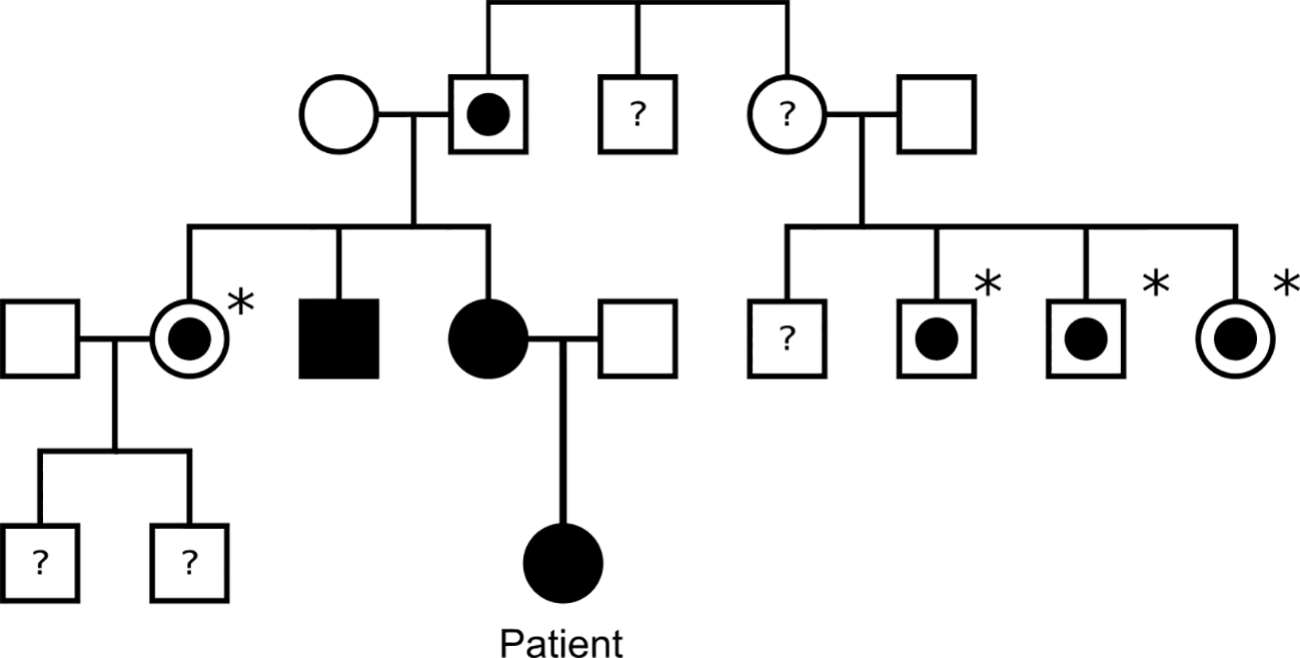
Family tree of the patient’s family. Black square/circle: male/female with paraganglioma and confirmed *SDHB* mutation. White square/circle with circle within: male/female with confirmed *SDHB* mutation, but no known paraganglioma. Asterisk: indicates patient has not undergone tumour screening. White square/circle: male/female with no known *SDHB* mutation or paraganglioma. Question mark: indicates patient has not undergone genetic testing for *SDHB*.

Germline genetic analysis confirmed a pathogenic *SDHB* variant c.136C>T p. (Arg46*) and predictive testing has identified several *SDHB* carriers on the maternal side of the family (**Figure 2**). Paired Whole Genome Sequencing (PWGS) detected somatic loss of the short arm of chromosome 1 (1p), consistent with loss of heterozygosity at the SDHB locus.

**Figure 3:**
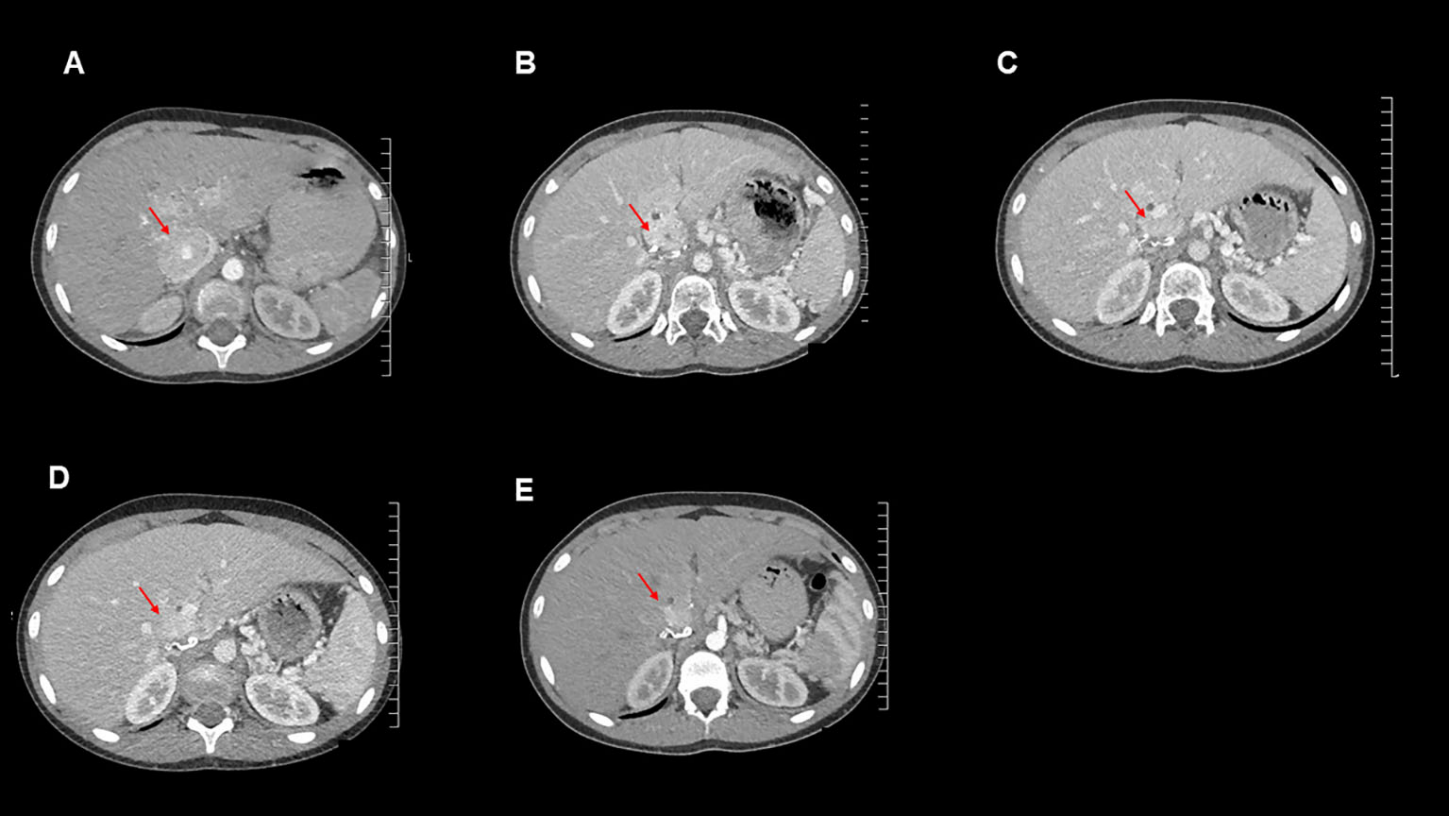
Axial contrast-enhanced CT images for this patient from the following time points: **(A)**: September 2020 (post operation), **(B)**: October 2021 (temozolomide therapy commenced), **(C)**: January 2022, **(D)**: April 2022, **(E)**: July 2022.

Over the subsequent 13 months, the patient remained largely asymptomatic, though she became tachycardic towards the end of this period and her plasma normetanephrine levels continued to increase (**Table 1**). She underwent normal pubertal development, achieved menarche and grew in height: she is now at the 9^th^ centile and still growing. Her alpha-blockade with doxazosin was restarted, initially at 1 mg once daily, then subsequently increased to 4 mg per day. Additionally, surveillance CT imaging showed evidence of disease progression (**Figure 3**).

Due to the patient’s biochemical and radiological progression, consideration of systemic therapy was discussed at an MDT meeting. An ^123^I-metaiodobenzylguanidine (MIBG) single positron emission computerised tomography (SPECT-CT) scan was carried out (**Figure 1G**), but showed poor ^123^I-MIBG uptake, indicating that the patient would be unlikely to benefit from MIBG therapy. The ^68^Ga-DOTATATE PET-CT was repeated (**Figure 1F**) to evaluate whether the patient’s metastatic nodal disease would be suitable for peptide receptor radionuclide therapy (PPRT) with ^177^Lu-DOTATATE, but due to heterogeneous uptake, this was not deemed the best option.

To assess the likely response of the tumour to temozolomide (1), O6-methylguanine methyltransferase (MGMT) methylation studies were undertaken and demonstrated MGMT promoter hypomethylation (mean methylation 2%) in the patient’s tumour cells. Despite this, temozolomide was still agreed as the best therapeutic option by the MDT. There is a good evidence base for the tolerability and safety of temozolomide in children (2, 3) and the toxicity profile is favourable to conventional chemotherapy regimens such as cyclophosphamide, vincristine, dacarbazine (CVD) (1). In addition, temozolomide was thought to be less likely to induce significant pubertal delay, and given the patient’s relatively low tumour burden was believed to have sufficient efficacy in this case.

Oral temozolomide was commenced in cycles of 5 days every 28 days, at a dose of 150 mg/m^2^/day for the first cycle and 200 mg/m^2^/day for each subsequent cycle, taken with ondansetron. This was initially well-tolerated by the patient, however in later cycles the patient suffered significant nausea, which was subsequently managed with dexamethasone and levomepromazine. In addition, the patient had several episodes of mild thrombocytopenia, necessitating total cumulative delays of 5 weeks over the 10 cycles, and dose reductions to 65% and 75% on three and two occasions respectively.

Imaging and measurement of plasma metanephrines were performed at 3-monthly intervals. These showed reduction in plasma metanephrine levels (**Table 1**) and decreases in size of the metastatic lesions on surveillance CT scans, indicating a response to temozolomide therapy (**Figure 3**). The MDT intend to continue to treat with temozolomide as long as the patient tolerates the drug and continues to improve clinically and biochemically.

## Discussion

### Phaeochromocytomas and paragangliomas in children

Phaeochromocytomas and paragangliomas (PPGL) are neuroendocrine tumours arising from neural crest cells. These can be functional (catecholamine-secreting) or non-functional. The term phaeochromocytoma refers exclusively to tumours arising within the adrenal medulla, whilst tumours arising in sympathetic and parasympathetic paraganglia elsewhere in the body are referred to as paragangliomas. PPGL are rare tumours in children, with an estimated overall incidence of 0.6 per 100,000 person-years (4). Of these, around two thirds are extra-adrenal and a third are adrenal (5). In addition, the prevalence of metastatic PPGL in children is significantly higher than in adults, comprising around half of cases (5, 6). PPGL typically present with signs and symptoms attributable to catecholamine excess, e.g. hypertension, flushing, sweating, headaches or palpitations (7). They may also present as an incidental finding on cross-sectional imaging or with symptoms of pain due to mass effect (8).

### Familial PPGL and *SDHB* mutations

Up to 80% of paediatric PPGL cases are thought to have a hereditary basis (5). These include hereditary tumour syndromes such as neurofibromatosis type 1, multiple endocrine neoplasia types 2A and 2B, and von Hippel-Lindau syndrome (9). Germline pathogenic variants in one of the genes encoding the succinate dehydrogenase complex (*SDHx*) also predispose to the development of PPGL. Such pathogenic variants are autosomal dominant, and require biallelic inactivation for tumorigenesis to occur (10). Higher levels of succinate in *SDHx*-deficient cells lead to increased stabilisation of hypoxia-inducible factor 1α (HIF-1α), as well as DNA hypermethylation. This can lead to neoplasia by driving angiogenesis, anaerobic metabolism and promoting hypermethylation (11).

The succinate dehydrogenase complex is made up of 4 subunits encoded by individual genes (*SDHA*/*B*/*C*/*D*). Of these, pathogenic variants in the *SDHB* gene confer the highest malignant potential, such that the majority (80%) of metastatic PPGL in children occur in patients with *SDHB* variants (12). *SDHB* variants more commonly lead to paragangliomas than phaeochromocytomas. Furthermore, they are implicated in the development of renal cell carcinoma (RCC), gastrointestinal stromal tumours (GISTs) and pituitary adenomas (13).

### Functional imaging modalities in PPGL

Several functional imaging modalities based on PET or SPECT are used for diagnosis and to guide therapy in PPGL. ^68^Ga-DOTATATE PET-CT is thought to be the most sensitive modality for imaging PPGL (14), especially for the detection of metastases (15). In addition, positive uptake of ^68^Ga-DOTATATE can indicate amenability to therapy with somatostatin receptor analogues, or peptide receptor radionuclide therapy with ^177^Lu-DOTATATE (16). Cases of absent or heterogeneous ^68^Ga-DOTATATE uptake, as noted here, have been reported in more aggressive *SDHx*-deficient PPGL and is hypothesised to reflect a degree of tumour dedifferentiation and loss of SSTR expression (14).

PET-CT can also be carried out using ^18^F-FDG, a tracer which acts as a marker of glucose metabolism. This is useful for assessing the metabolic activity of PPGL or suspected metastases, but is less sensitive than ^68^Ga-DOTATATE imaging for detecting metastases in patients with secretory PPGL because of brown fat activation by catecholamine secretion and ^68^Ga-DOTATATE PET/CT is a more specific tracer for PPGL (17).

^123^I-MIBG is a tracer which binds to the noradrenaline transporter and can be used in conjunction with SPECT-CT imaging. Its sensitivity is also inferior to ^68^Ga-DOTATATE in detecting metastatic disease (14), and particularly in *SDHx*-deficient PPGL (15). It nonetheless remains a useful theranostic imaging modality, predicting whether a tumour may be suitable for treatment with to ^131^I-MIBG therapy.

### Therapeutic options in metastatic PPGL

In addition to symptom management with alpha- and beta-blockers, there are several therapeutic approaches for metastatic PPGL. Radionuclide therapy with ^131^I-MIBG or ^177^Lu-DOTATATE are commonly used (18). Both these approaches are only possible if respective functional imaging modalities show sufficient uptake of analogous tracers (see above).

The first-line cytotoxic chemotherapy regime in metastatic PPGL has traditionally been cyclophosphamide, vincristine, and dacarbazine (CVD) (18). Of these, dacarbazine has been hypothesised to be the only active agent against PPGL (19). Dacarbazine is converted to an active alkylating agent, methyl-triazene-1-yl-imidazole-4-carboxamide (MTIC), which is also the active metabolite of temozolomide (20). Temozolomide is thought to have favourable pharmacokinetics and a better toxicity profile when compared to dacarbazine (21), whilst having similar efficacy (22).

Two small studies (19, 20) and several case reports (23) have found temozolomide to be an effective chemotherapy in a number of patients with metastatic PPGL. Reported response to temozolomide was shown to be greater if the patient had a germline *SDHB* mutation (19, 20). When used for glioblastoma multiforme (GBM), the efficacy of temozolomide is known to be correlated with the methylation status of the MGMT promoter: hypermethylation of the MGMT promoter is associated with a better response, likely due to a decreased ability of tumour cells to repair damage to DNA (24). This association has also been borne out by a small study of the use of temozolomide in metastatic PPGL, in which 80% of responders to temozolomide therapy had MGMT promoter hypermethylation. In addition, the study also found a correlation between *SHDB* mutations and MGMT promoter hypermethylation (19), suggesting one possible mechanism by which *SDHB* mutations may confer susceptibility to temozolomide.

### Use of temozolomide in children with metastatic phaeochromocytoma/paraganglioma

Various options for systemic therapy were considered in this case. The mainstay of chemotherapy for PPGL has traditionally been CVD (cyclophosphamide, vincristine, dacarbazine), but this is associated with significant side effects such as myelosuppression, peripheral neuropathy and gastrointestinal toxicity (25). Systemic radionuclide therapy using ^131^I-MIBG or ^177^Lu-DOTATATE is also possible, but only if functional imaging has confirmed sufficient tracer uptake (see introduction). ^131^I-MIBG is an effective palliative treatment for PPGL (26), but can cause severe haematological toxicity (27). If the tumour is SSTR positive, PPRT with ^177^Lu-DOTATATE is an option. This has reduced haematological toxicity compared to ^131^I-MIBG therapy, and necessitates shorter inpatient stays (16). In SSTR-positive cases, there may also be a role for combination or maintenance therapy with somatostatin analogues such as octreotide or lanreotide (26).

There are few published reports on the use of temozolomide in children with PPGL, with the current evidence base being limited to two case reports in which temozolomide was trialled in the treatment of adolescents with metastatic paragangliomas alone (28) and in combination with olaparib (29). A more recent study of temozolomide use in metastatic *SDHx*-deficient PPGL includes a child aged 13 within its cohort (20). There are several reports of the use of temozolomide in adults with PPGL which point particularly towards the benefits of temozolomide therapy in *SDHB*-deficient PPGL (19, 20, 23). The tolerability of temozolomide in these patients was also thought to be superior to standard CVD therapy, with few patients experiencing adverse effects (19, 23). There is also a good evidence base for temozolomide being well-tolerated in other paediatric cancers such as GBM (2).

The partial response to temozolomide means that this case adds to existing literature by showing that temozolomide can also be an effective treatment option for a metastatic *SDHB*-related paraganglioma in a child. Moreover, the increased tolerability of temozolomide in children compared to standard chemotherapy regimens such as CVD may make the risk-benefit profile preferable to these agents, particularly during the adolescent period when consideration also needs to be given to growth and puberty, in addition to education and overall psychological wellbeing. Indeed, a key consideration in the management of this case was the timing of systemic chemotherapy. Alkylating agents such as temozolomide are linked to premature ovarian failure and pubertal delay (30, 31), therefore a balance had to be struck between the benefits of chemotherapy, and allowing the patient to grow and undergo puberty. The onset of temozolomide therapy was therefore delayed to minimise these effects and was eventually commenced 15 months after diagnosis. Cryopreservation was not performed in this case, as the patient was not eligible based on local criteria.

Given the lack of evidence around its use in children with PPGL, the optimal duration of treatment with temozolomide is unclear. Most of the data surrounding the use of temozolomide concerns its use as an adjuvant to radiotherapy in the treatment of GBM (32), whereas in this case its use is palliative. An Anglo-French study of temozolomide monotherapy for GBM in children used up to 24 cycles of chemotherapy, and found that haematological toxicity was the main side-effect, with myelosuppression necessitating delays and dose reductions in 17% and 22% of all cycles respectively (3). In this case, thrombocytopenia delayed several cycles and caused dose reductions, reflecting these findings. Ongoing therapy will therefore need to balance the impact of temozolomide on disease progression against potential toxicity and reduced tolerability.

## Conclusion

This report demonstrates that temozolomide therapy can be an effective therapeutic option for children with *SDHB*-deficient metastatic paraganglioma. Further work is needed however, to establish whether temozolomide is preferable to standard chemotherapy with CVD in children. The case also identifies a potential pitfall in the use of SSTR functional imaging in metastatic paraganglioma, in that metastatic lesions may be missed if there is dedifferentiation and loss of SSTR expression.

## Data availability statement

The original contributions presented in the study are included in the article/supplementary material. Further inquiries can be directed to the corresponding author.

## Ethics statement

Written informed consent was obtained from the minor(s)’ legal guardian/next of kin for the publication of any potentially identifiable images or data included in this article.

## Author contributions

CU drafted and revised the initial manuscript, **Figure 2** and **Table 1**. RC, AH and JN helped supervise the writing process and edit the manuscript. VK, CJ and A-ML provided input about the operative aspects of the case. BF, IH, MM, LA and FG contributed information and figures from the imaging undertaken in the case, and helped edit the manuscript. RA and PT provided input about the genetics of the case. LH provided information about the histopathology of the case. All authors contributed to the article and approved the submitted version.

## Funding

Ruth Casey obtained funding from GIST Support UK.

## Conflict of interest

The authors declare that the research was conducted in the absence of any commercial or financial relationships that could be construed as a potential conflict of interest.

## Publisher’s note

All claims expressed in this article are solely those of the authors and do not necessarily represent those of their affiliated organizations, or those of the publisher, the editors and the reviewers. Any product that may be evaluated in this article, or claim that may be made by its manufacturer, is not guaranteed or endorsed by the publisher.

## Abbreviations

CVD, cyclophosphamide, vincristine, dacarbazine; FDG, fluorodeoxyglucose; GBM, glioblastoma multiforme; MIBG - metaiodobenzylguanidine; MDT, multidisciplinary team; MRI, magnetic resonance imaging; MTIC, methyl-triazene-1-yl-imidazole-4-carboxamide; PET, positron emission tomography; PPGL, phaeochromocytoma/paraganglioma; PPRT, peptide receptor radionuclide therapy; *SDHB*, succinate dehydrogenase complex subunit B; *SDHx*, succinate dehydrogenase; SPECT, single-photon emission computerised tomography; SSTR, somatostatin receptor.
